# The Interplay between Estrogen and Fetal Adrenal Cortex

**DOI:** 10.1155/2012/837901

**Published:** 2012-03-28

**Authors:** Jovana Kaludjerovic, Wendy E. Ward

**Affiliations:** ^1^Department of Nutritional Sciences, Faculty of Medicine, University of Toronto, Toronto, ON, Canada M5S 3E2; ^2^Department of Kinesiology, Center for Bone and Muscle Health, Faculty of Applied Health Sciences, Brock University, St. Catharines, ON, Canada L2S 3A1

## Abstract

Estrogen is a steroid hormone that regulates embryogenesis, cell proliferation and differentiation, organogenesis, the timing of parturition, and fetal imprinting by carrying chemical messages from glands to cells within tissues or organs in the body. During development, placenta is the primary source of estrogen production but estrogen can only be produced if the fetus or the mother supplies dehydroepiandrosterone (DHEA), the estrogen prohormone. Studies show that the fetal zone of the fetal adrenal cortex supplies 60% of DHEA for placental estrogen production, and that placental estrogen in turn modulates the morphological and functional development of the fetal adrenal cortex. As such, in developed countries where humans are exposed daily to environmental estrogens, there is concern that the development of fetal adrenal cortex, and in turn, placental estrogen production may be disrupted. This paper discusses fetal adrenal gland development, how endogenous estrogen regulates the structure and function of the fetal adrenal cortex, and highlights the potential role that early life exposure to environmental estrogens may have on the development and endocrinology of the fetal adrenal cortex.

## 1. Introduction

The fetal-origin hypothesis put forth by Dr. David Barker nearly three decades ago challenged the traditional views on the pathogenesis of common chronic diseases [1]. The Fetal-Origin Hypothesis, later termed the Developmental Origin of Health and Disease, established the principle that perinatal events are memorized by the developing organism through fetal and neonatal imprinting [2]. In light of this hypothesis, perinatal events can be thought of as the foundation for structural and functional development of an organism. All organisms arise from a unique DNA sequence that gives rise to specialized cell phenotypes through well-regulated gene expression and epigenetic regulation [3]. Each cell phenotype can be influenced by its internal and external environment. In the human body, there are approximately 200 specialized cells that are influenced by internal and external steroid hormones during development [3].

Steroid hormones act as chemical messengers to induce both slow genomic and rapid nongenomic responses and, thus, modulate a wide array of essential cellular and physiological responses. In the initial stages of pregnancy, steroid hormones send signals that allow the embryo to successfully implant in the posterior wall of the uterus and regulate cell proliferation, differentiation, and gene transcription. In the later stages of gestation, steroid hormones support fetal growth and development by modulating metabolic processes of embryogenesis and organogenesis. Steroid hormones also play a pivotal role in regulating the timing of parturition. Different steroid hormones are expressed at different stages of intrauterine life suggesting that temporal and spatial expression of steroid hormones is critical for fetal growth and development [4, 5]. Of all the steroid hormones produced during pregnancy, estrogen has attracted much interest because it is expressed at every stage of gestation and modulates many intrauterine processes.

Estrogen synthesis takes place in the ovaries, adrenal cortex, and the placenta. During pregnancy, the placenta becomes the primary site of estrogen synthesis; however, placental estrogen production can only be achieved through input from fetal and/or maternal adrenal cortex. This is because of the placenta's inability to produce the androgenic C_19_ steroid (dehydroepiandrosterone, DHEA, and its sulfoconjuate, DHEA-S), the essential substrate for placental estrogen [4, 6, 7]. The fetal adrenal gland provides a larger proportion of the byproducts used for placental estrogen production [8]. Appropriate development and function of the fetal adrenal cortex is therefore critical for placental steroid production (i.e., estrogen, cortisol, and aldosterone), fetal maturation, and perinatal survival [9].

Emerging studies suggest that environmental estrogens can disrupt the natural interplay between estrogen and functional biology of the fetal adrenal cortex [10, 11]. Humans are exposed daily to environmental estrogens through the food supply and the use of pesticides, herbicides, petroleum byproducts, and plastics. Some examples include soy isoflavones, bisphenol A, DDT, polychlorinated biphenyls, polybrominated diphenyl ethers, and a variety of phthalates. Although environmental estrogens pose minimal threat to adults, they may have an adverse effect on human health if exposure occurs during critical stages of development when cells are more easily influenced by steroid hormones [2]. Most of the supporting studies in this area have focused on a synthetic estrogen, diethylstilbestrol (DES). From about 1940–1970, DES was prescribed to pregnant women based on the belief it could prevent miscarriage (it is no longer used because of adverse effects, particularly to offspring). In vitro assays have shown that compared to 17*β*-estradiol the estrogenic potency of DES is 0.5 and that of all other environmental estrogens listed previously is <0.0001 [12, 13]. Based on the estrogenic potency, the adverse effects induced by DES may be more severe than those induced by environmental estrogens and thus have more subtle effects that are nonetheless disrupting placental estrogen production and fetal development. This paper examines the available literature on fetal adrenal gland development, how endogenous estrogen regulates the structure and function of the fetal adrenal cortex, and highlights the potential role that early life exposure to environmental estrogens may have on the development and endocrinology of the fetal adrenal cortex.

## 2. Estrogen Synthesis in Utero

Pioneering work by Edward Adelbert Doisy revealed that three forms of estrogen (estradiol, estriol, estrone) are present in the urine of pregnant women [15]. These three forms of estrogen have also been detected in the fetal circulation throughout gestation and in the umbilical cord plasma at birth [16]. Concomitantly, the rate of estrogen production and the amount of estrogen in the maternal and fetal plasma increases markedly throughout pregnancy. By term, estradiol and estrone levels are 100-fold higher than those of nonpregnant women and estriol levels are up to 1000-fold higher [8]. Although these high concentrations of estrogen are destined for maternal compartments, they also induce a number of biological effects on the developing fetus (Table 1).

**Figure 1:**
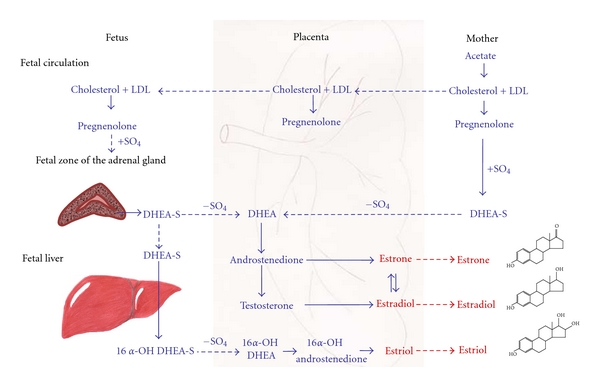
The role of maternal, placental, and fetal units in the biosynthesis of estrone, estradiol, and estriol. (LDL: low-density lipoproteins; DHEA-S: dehydroepiandrosterone sulfate; OH: hydroxyl). This figure has been modified from [8, 14].

During early stages of pregnancy, estrogen is synthesized by the corpus luteum in the maternal ovaries and its primary function is to ensure that the mother does not reject the developing embryo. To accomplish this, estrogen enhances myometrial activity, softens collagen fibers in the cervical tissue, and promotes vasodilation of the uterus. Around the 8th week of gestation when the fetoplacental unit has a highly vascularized network, placenta becomes the primary source of estrogen production [4]. However, the production of estrogen does not rely solely on the placenta. The placenta converts C_19_ steroids into estrogen but cannot produce estrogen de novo from acetate or cholesterol and cannot convert pregnenolone or progesterone into C_19_ steroids because it lacks the primary 17*α*-hydroxylase (P450c17) enzyme [4, 6, 7]. Estrogen synthesis by the primate placenta is therefore achieved through stereogenic interplay between the maternal-placental unit and the fetal-placental unit (Figure 1). In this system, the maternal-placental unit permits low-density lipoproteins (LDLs) and/or free cholesterol to pass through the placenta and enter the fetal circulation. The fetal adrenal cortex, which expresses high amounts of P450c17 enzyme, has a highly developed ability to bind and use cholesterol to produce the DHEA and DHEA-S, which are the essential substrates for placental estrogen production. Near term, 60% of the total DHEA-S used by the placenta for estradiol and estrone synthesis is derived from the fetal adrenal cortex, whereas only 40% is derived from the maternal adrenal cortex [8]. The fetus, unlike the mother, also produces 90% of 16*α*-hydroxyl DHEA-S, which is a substrate for estriol synthesis. Therefore, the fetus rather than the mother provides most of the byproducts used for placental estrogen production [8].

## 3. Fetal Adrenal Gland Development

**Figure 2:**
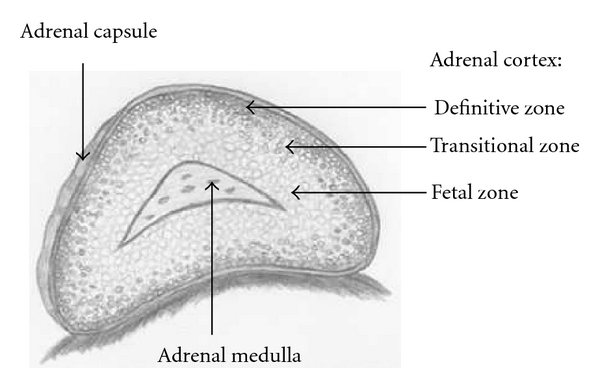
Illustration of the midgestation human fetal adrenal gland.

 Development of the fetal adrenal gland is a dynamic process that is characterized by rapid cell growth, unique morphological development, and high steroidogenic activity. Throughout gestation, the size and weight of the fetal adrenal gland continuously grow but, unlike most other organs, the adrenal gland regresses substantially from fetal to adult life [17, 18]. At birth, the fetal adrenal gland weighs between 3 and 4 grams and is 20 to 30 times larger than its adult counterpart [17–20]. Moreover, its size at birth is approximately equal to that of the fetal kidney [4]. Studies focusing on structural biology have revealed that the large size of the fetal adrenal gland depends on a single structural compartment called the fetal zone (Figure 2), identified by Elliott and Armour in 1911 [21]. The fetal zone occupies the central region of the fetal adrenal cortex and by mid-gestation accounts for 80–90% of the fetal adrenal gland [17, 22]. Functionally, the fetal zone resembles the adult zona reticulosa because it is the primary site of steroidogenesis [23]. Beginning around the 10th week of gestation, the fetal zone expresses P450c17 enzyme and produces DHEA, the precursor for placental estrogen production. Therefore, fetal zone is the primary site of estrogen synthesis during gestation [4]. Because placental estrogen production is exclusive to intrauterine life, the fetal zone begins to regress at birth and completely disappears in the first few months of life. The demise of the fetal zone induces postnatal remodeling and causes more than a 90% reduction in the adrenal gland size [23].

In addition to the fetal zone, the fetal adrenal cortex in utero is composed of two other morphologically distinct zones: the definitive zone and the transitional zone [9]. The definitive zone, which is also called the neocortex, develops as a narrow band of tightly packed cells surrounding the fetal zone. It expresses the enzyme 3*β*-hydroxysteroid dehydrogenase (3*β*-HSD) that produces aldosterone. Thus, it is believed to be the site that gives rise to the postnatal adrenal glomerulosa [23, 24]. The third zone, the transitional zone, develops in late gestation between the fetal and definitive zone and gives rise to zona fascicularis that produces glucosteroids [24, 25]. The formation and dynamics of the fetal zone, definitive zone, and transition zone form the fetal adrenal cortex. In turn, the adrenal cortex can have long-term effects on the developing organism.

## 4. Embryogenesis and Development of the Fetal Adrenal Gland

Embryonic development of the fetal adrenal gland involves cellular hyperplasia, hypertrophy, migration, and senescence [4, 19]. As early as five weeks of gestation, the migration of primitive cells accumulates at the cranial end of the mesonephros in what is called the “adrenal blastema” [19]. The adrenal blastema, which is thought to be the first identifiable manifestation of the adrenal gland, exhibits mitotic activity, suggesting that it develops by hyperplasia [4, 19, 26]. Thereafter, neighboring primordial cells which are destined to become steroidogenic cells are recruited to the adrenal blastema [27]. Interestingly, these primordial cells are the same cells that give rise to steroidogenic activity in the gonads [27]. Ultimately the migration of primordial cells from the primordial mesoderm determines whether these cells will commit to the adrenal gland or the gonads. Cells on the dorsal aortal side of the primordial mesoderm give rise to steroidogenic activity in the adrenal cortex, whereas those on the coelomic epithelial side migrate towards the gonads. The cells committed to the adrenal gland form a cord cluster with the adrenal blastema. The cord cluster cells can then either differentiate into large polyhedral cells that give rise to primordium of the fetal zone or can maintain their small morphology and form the definitive zone of the fetal adrenal cortex [4].

The large polyhedral cells have unique characteristics including a larger number of tubular smooth endoplasmic reticula, large Golgi complexes, and a greater number of mitochondria with well-defined cristae. These cells are also characterized by their high lipid production and abundance of highly dense bodies [4]. These cellular properties are consistent with characteristics of steroid-producing cells keeping in agreement that the fetal zone is the primary site of steroidogenesis in the fetal adrenal gland [4]. The cells in the fetal zone aside from having a unique structural composition are arranged in a specific manner that optimizes the production of DHEA-S. The cells in the central portion of the fetal zone are arranged far apart in a netlike pattern with many vascular sinusoids, while the cells on the outer region of the fetal zone are arranged in tightly packed cords [28]. This type of arrangement is designed to speed up the transfer of molecules through the cells and promotes steroidogenesis.

Comparative studies have shown that the cells in the definitive zone are two to five times smaller than those in the fetal zone. They are packed tightly together with small ribosomes, small dense mitochondria containing cristae, and a poor lipid profile [4]. Out of all these cellular characteristics it is the poor lipid profile that has the greatest biological effect as lipid composition modulates steroidogenic activity. Cells with a lipid-rich profile can produce steroidal compounds, whereas those that have a lipid-poor profile lack the fundamental building blocks required for steroidogenesis. The lipid-poor profile in the definitive zone of the adrenal gland is observed only in the first half of pregnancy. At midgestation, the definitive zone synthesizes aldosterone, a cholesterol byproduct, and thus, its cellular lipid profile improves around this time [28].

 Another unique property of the definitive zone is its mitotic activity. By 10–12 weeks of gestation, the definitive zone exhibits many mitotic subunits [19]. Thus, the definitive zone grows mainly by hyperplasia, whereas the fetal zone grows by hypertrophy [18, 19]. The rapid cell proliferation in the definitive zone causes mitotic pressure in the periphery of the adrenal cortex leading to centripetal migration of cells to the fetal zone [19, 20]. Work by Morley et al. [29] showed that in a mouse embryo the centripetal invasion of cells is only established in the later stages of the fetal life when the lipid composition of cells is improved. This centripetal migration lends support to the migration theory of the adrenal cortical cytogenesis and suggests that the definitive zone is the stem cell compartment from which the inner cortical zones are developed [4]. It is hypothesized that soon after birth, the fetal zone disappears due to increased apoptosis in this region and the centripetal migration of cells from the definitive zone drives the development of zona glomerulosa and fasciculate [19, 30].

 The adrenal medulla which is composed of hormone-producing chromaffin cells does not exist as a discrete structure throughout gestation. Instead it is composed of small islands of chromaffin cells that in the first few weeks of gestation are scattered throughout the fetal adrenal cortex but then aggregate in the fetal zone. The disappearance of the fetal zone at the beginning of postnatal life stimulates the already aggregated chromaffin elements around the central vein to form elementary medulla. This elementary medulla undergoes extensive remodeling, and by the fourth week of postnatal life, almost all of the chromaffin cells are clustered in the central region of the adrenal gland. However, it is not until one to two years postpartum that the morphological development of the adrenal medulla resembles its adult counterpart [4]. Further research is needed to determine whether disruption of fetal adrenal cortex has an effect on the development and function of the adrenal medulla.

The development of the fetal adrenal gland is a well-orchestrated phenomenon that requires tight regulation. Significant progress has been made in elucidating the role of steroidogenic enzymes and nuclear receptors, fetal and maternal hypothalamic-adrenal function, and placental estrogens on fetal adrenal function, growth, and development.

## 5. The Role of Steroidogenic Enzymes and Nuclear Receptors in Differentiation of Cortical Zone Function

The steroidogenic potential of fetal cortical zones depends on its steroidogenic enzymes and nuclear receptors. The two primary steroidogenic enzymes of the fetal adrenal gland are P450c17 and 3*β*-HSD [4, 23]. Findings from immunohistochemistry have revealed that P450c17 enzyme is expressed in the fetal zone, 3*β*-HSD is expressed in the definitive zone, and that both enzymes are expressed in the transition zone [4, 31]. These patterns of enzyme expression provide critical clues regarding the steroidogenic role of each fetal adrenal zone. The P450c17 enzyme stimulates DHEA-S production, the 3*β*-HSD enzyme drives aldosterone production, and the combined effects of both enzymes are responsible for cortisol synthesis [4]. Based on this evidence and on the in situ patterns of cholesterol cleavage, it has been determined that the fetal zone, definitive zone, and transition zone are sites of DHEA-S, aldosterone, and cortisol synthesis, respectively [4]. For most of human pregnancy, the fetal adrenal cortex lacks the 3*β*-HSD enzyme inhibiting its cortisol and/or aldosterone production. Therefore, the cholesterol substrate is used primarily for DHEA-S production, making it the primary byproduct of the fetal adrenal gland during gestation [32, 33].

 According to human experiments, radiolabeled DHEA-S can be converted to estradiol when perfused through the placenta [34]. Human placenta produces estrogen by the aromatization of DHEA and its 16*α*-hydroxylated metabolite (16*α*-OH DHEA) that is produced by the combined efforts of the adrenal cortex and the liver [4, 35]. In the placenta, 3*β*-HSD converts DHEA to androstenedione. Thereafter, the androstenedione can either be converted to estrone by an aromatase enzyme or to testosterone by 17*β*-hydroxysteroid dehydrogenase (17*β*-HSD). The newly synthesized estrone and/or testosterone can be further converted to estradiol by 17*β*-HSD or aromatase enzyme, respectively (Figure 1).

## 6. The Relationship between Fetal and Maternal Hypothalamic-Adrenal Function

The ongoing interaction between the fetus and the mother has been the focus of many studies as scientists strive to understand the factors responsible for maintaining pregnancy and stimulating labor and delivery. The essential and universal component of pregnancy is the endocrine communication between the mother and the fetus which greatly depends on the maternal and fetal adrenal cortex. To understand the specific role of fetal and maternal adrenal gland function during gestation, two adrenoectomized models have been developed: the maternal adrenalectomy model and the fetoectomy model. In the first model, which is usually performed in rodents, the adrenal glands are surgically removed from the mother during gestation while the placenta and the fetus are kept in situ. In the second model (i.e., fetoectomy), which is typically performed in primates, the fetus is surgically removed from the uterus and the placenta is kept in situ. The use of these two experimental models has helped scientists uncover some information about the interaction between the maternal and fetal adrenal gland function.

**Figure 3:**
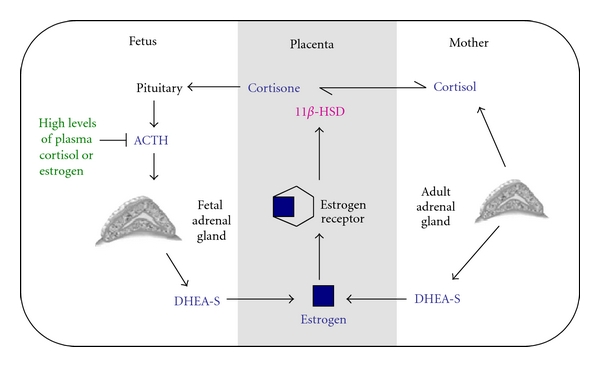
Representation of how placental estrogen during gestation modulates its own production. Placental estrogen has a positive and a negative feedback mechanism to ensure that physiological levels of estrogen are established. Estrogen produced by the placenta induces a positive feedback mechanism by promoting the conversion of cortisol to its biologically inactive metabolite cortisone through upregulation of 11*β*-HSD expression. Serum cortisone levels stimulate the fetal pituitary gland to produce ACTH which upregulates DHEA-S production from the fetal adrenal cortical zone and leads to placental estrogen production. In contrast, if placental estrogen concentrations are too high, placental estrogen can inhibit its own production by suppressing the responsiveness of the fetal adrenal zone to ACTH and lowering its production of DHEA-S. This figure has been modified from Albrecht et al. [36].

Studies examining maternal adrenalectomy have revealed that the absence of maternal adrenal gland causes a substantial reduction in plasma cortisol and estrogen levels. These endocrine disruptions activate the fetal pituitary gland to secrete adrenocorticotropic hormone (ACTH) [37–39]. Upregulation of ACTH promotes fetal production of corticosteroids (i.e., DHEA and DHEA-S) from the fetal adrenal cortex, which serve as substrates for placental endocrine production (Figure 3). Under these conditions, the fetus is placed under stress and its fetal adrenal cortex takes over at least part of the maternal adrenal function. In a study investigating Sprague-Dawley rats, plasma DHEA-S levels of adrenalectomized dams rose from very low levels at 18 days of gestation to levels that were substantially higher than those of sham rats on 21 days of gestation [40]. This finding suggests that there is a critical factor that compensates for the lack of maternal corticosteroid production. Based on the available evidence, we suspect that the fetal adrenal cortex is the primary modulator of these effects. However, further studies need to be conducted to support this hypothesis. Moreover, research is required to determine whether maternal adrenalectomy has long-term effects on offspring's neuroendocrine system that controls reaction to stress, digestion, immune function, mood and emotion, sexuality, energy storage, and/or expenditure.

The endocrine effects of fetoectomy have only been partially elucidated. Studies in baboons and rhesus monkeys have shown that fetoectomy—a process where the fetus is removed and the placenta is kept in situ—alters the maternal hypothalamic-pituitary-adrenal axis [41–43], eliminates the 24-hour steroid rhythm [44], and causes a loss in uterine activity [44]. Moreover, removal of the fetal adrenal gland during late gestation has been shown to cause a significant reduction in maternal plasma ACTH levels and a concomitant decrease in circulating cortisol and DHEA-S levels [41]. Interestingly, the drop in plasma DHEA-S concentrations between intact and fetoectomized animals was roughly 35% [41], which is equivalent to the difference between pregnant and nonpregnant animals [45]. Under normal physiological conditions, a drop in DHEA-S production is directly correlated with a decline in maternal estradiol and progesterone concentrations. This too was observed in fetoectomized baboons [42, 46] and monkeys [41, 43, 44]. Reintroducing DHEA-S to the placenta after fetoectomy was shown to restore placental estradiol production [46]. This data indicates that fetoectomy does not modulate placental function but rather that it affects the production of DHEA substrates needed by the placenta to generate endocrine hormones. Thus, estrogen production in the maternal circulation is an indicator of fetal adrenal steroidogenic activity. Taken together, this data strongly supports the idea that fetal adrenal cortex plays a primary role in modulating the feto-maternal endocrine communication.

## 7. Estrogen-Mediated Effects on Fetal Adrenal Function

 The discovery that estrogen receptor alpha (ER-*α*) and beta (ER-*β*) are expressed in all zones of the fetal adrenal cortex suggests estrogen has a direct effect on fetal adrenal function. By binding to ER-*α* and ER-*β*, estrogen can induce both direct and indirect effects on the fetal adrenal cortex, and depending on plasma estrogen levels, it can stimulate or inhibit its cellular responsivity to ACTH [36]. As depicted in Figure 3, when high quantities of plasma estrogen are reached, estrogen acts directly on the fetal adrenal cortex to suppress its responsivity to ACTH activity. This inhibits DHEA-S production from the fetal zone of the adrenal cortex and thereby lowers placental estrogen synthesis [36, 47, 48].

 Comparative real-time PCR studies have shown that ER-*α*, ER-*β* mRNA, and protein expression is increased from mid- to late gestation in the definitive and transitional zones of the fetal adrenal gland, but not in the fetal zone [49, 50]. By lowering the number of available ERs on the fetal zone of the adrenal gland, the negative feedback system that suppresses DHEA-S production is desensitized, the estrogen, mediated positive feedback system is activated, and production of DHEA-S is upregulated (Figure 3).

 Long-term cultures of human fetal adrenal cells have shown that estrogen binding to ER-*α* and ER-*β* can stimulate DHEA-S production through an indirect pathway that suppresses cortisol synthesis [51]. Cortisol is a stress hormone that maintains intrauterine homeostasis and influences the development and maturation of many fetal tissues including the lungs, liver, intestine, and the central nervous system [52]. Too much cortisol during pregnancy may have an adverse effect on fetal development by increasing maternal stress and blood pressure [52]. To protect the fetus from too much cortisol, the placental enzymes, 11*β*-hydroxysteroid dehydrogenase (11*β*-HSD), convert cortisol to the biologically inactive steroid, cortisone [4, 36, 53, 54]. This placental enzyme is regulated by estrogen and progesterone [55]. An increase in either estrogen or progesterone stimulates 11*β*-HSD to increase transplacental oxidation of cortisol to cortisone [55]. Low cortisol levels stimulate secretion of ACTH by the fetal pituitary gland, which in turn stimulates DHEA-S production from the fetal cortical zone, providing more substrate for placental estrogen production. In cases where ACTH levels become too high, the estrogen feedback loop may be turned off by stimulating cortisol synthesis (Figure 3) [36].

 In late gestation, ACTH receptor mRNA expression is higher in the definitive and transitional zones than in the fetal zone of the human fetal adrenal cells. This suggests that ACTH promotes cortisol production during late gestation. Cortisol can inhibit estrogen synthesis by suppressing ACTH activity from the fetal pituitary gland. However, in late gestation, this pathway is likely not activated because high amounts of estrogen are required for initiation of parturition. Instead, high plasma cortisol levels in late gestation are likely converted to cortisone through the estrogen-mediated positive feedback system (Figure 3).

 The available data has provided much mechanistic insight regarding the role of estrogen in modulating the development and function of fetal adrenal cortex [4, 36]. However, to more fully understand the role of estrogen in the fetal adrenal gland, it is important to better characterize the expression of coactivators and corepressors as well as the pattern of ER heterodimerization in different regions of the fetal adrenal gland. Moreover, three estrogen isoforms have been identified but the precise role of these isoforms in the physiology of pregnancy has not been elucidated. This may be one of the limiting factors in our current understanding of estrogen and fetal adrenal development. It is possible that structural differences between the three estrogen isoforms serve to induce selective effects at the level of nuclear receptor. Future research in this area will need to decipher the role of estradiol, estrone, and estriol on adrenal gland development and function.

## 8. Estrogen-Mediated Effects on Fetal Adrenal Growth and Development

Only one study has examined the effects of plasma estrogen levels on developmental differentiation of fetal cortical zones [11]. In this paper, fetal adrenal glands were obtained from baboons that had normal plasma estrogen levels and from those that had abnormally low estrogen levels due to administration of a highly specific aromatase inhibitor. Findings from this study revealed that fetal adrenal weight and volume increased 3-fold between mid- and late gestation and an additional 70% by aromatase administration, which decreased serum estradiol levels by 95%. The 70% rise in fetal adrenal growth among estrogen-deprived baboons was explained by a significant increase in the fetal zone. It was the only adrenal cortical site that exhibited any change in weight. This is an interesting finding because although ER-*α* and ER-*β* are expressed in all zones of the fetal adrenal cortex [36], loss of estrogen had a selective effect on the fetal zone, the primary site of stereocorticoid production. The observed increase in fetal zone size among estrogen-deprived baboons was associated with a 3-fold rise in fetal serum DHEA-S levels. Based on this evidence, researchers from this study concluded that plasma estrogen concentrations selectively repress both the morphological and functional development of the fetal zone in utero between mid- and late gestation [11]. According to these findings, it is possible that estrogen produced by the placenta feeds back to the fetal zone of the fetal adrenal cortex to restrain its growth and development. By controlling the size of the fetal zone, estrogen may be able to regulate the amount of DHEA-S being produced and, in turn, its own production. This is important because having high levels of estrogen can lead to endocrine disruption that may have deleterious effects on fetal growth and maturation [56].

## 9. Does Exposure to Environmental Estrogens Affect the Fetal Adrenal Gland Development and Function?

 Exposure to environmental estrogens during perinatal life may have permanent long-lasting consequences on the overall growth and development of the fetus [56]. The fetus has a faster metabolic rate, a weaker immune system, and a lower concentration of detoxifying enzymes and liver metabolites compared to an adult, making it more sensitive to environmental disruptions [57]. Moreover, due to its small body size, exposure to environmental estrogens may cause chemical toxicity in the fetus at a concentration that has no adverse effect in an adult [57]. A growing number of commercially available products containing low doses of estrogen also increase the risk of estrogen accumulation in the mother as well as the fetus. Fetal cells are highly susceptible to change, so exposure to environmental estrogens, powerful chemical messengers, may induce adverse effects on fetal growth, development, and future health.

Although there are many environmental estrogens in the world today, their effects on fetal programming as well as fetal adrenal gland development and function have not been extensively studied. One exception is DES, a synthetic estrogen that used to be administered to pregnant women for prevention of miscarriages from the mid-1940s to 1970s [58–60]. Its use was discontinued, after several million offspring were exposed, because it was discovered that in utero exposure to DES causes major developmental changes in the offspring. It disrupts reproductive tract development and function at sexual maturity in 30% of the exposed population [61, 62]. Female offspring exhibit reduced fertility and significant increase in vaginal carcinomas, while male offspring suffer from reduced testicular size and sperm count [57]. Both genders, when exposed to DES during prenatal development, are susceptible to benign tumors in reproductive organs and autoimmune disorders [57]. Therefore, developmental changes induced by in utero exposure to DES have long-term programming effects on reproductive function. As previously mentioned, the primordial cells that give rise to reproductive organs are the same cells that give rise to the fetal zone of the fetal adrenal gland [4] (see Section 4), suggesting that prenatal exposure to DES may also disrupt adrenal gland development and function. Female rats treated with DES from gestation day 8 to 18 had a 30% enlargement in adrenal gland weight and a significantly lower concentration of circulating adrenal steroid hormones compared to the control group, including a 60% and 32% reduction in serum progesterone and estrogen levels, respectively [63]. The exposure to DES was also shown to reduce uterine contractions, prevent placental detachment from the uterine wall, and increase fetal death.

In examining fetal survival, it appears that the rat fetus is the most sensitive to DES treatment when exposure occurs between 18 and 20 days of gestation [63], suggesting that exposure to environmental estrogens has the most profound effect on fetal development in late gestation. This is not surprising because DES is structurally similar to estradiol and, as such, can bind to ERs to induce estrogen-like effects [64, 65]. Exposure to exogenous estrogens during late gestation has the potential to activate the negative feedback loop to inhibit DHEA-S production from the fetal adrenal cortex because the fetus is already exposed to high levels of endogenous estrogen. Moreover, such high levels of estrogen could cause a decrease in blood gonadotropins (i.e., FSH, LH) which in turn could slow down follicular development and depress follicular synthesis of progesterone and testosterone [66–68]. The aromatase enzyme converts testosterone to estradiol, and thus, this pathway may also be turned off [69]. Depressed fetal zone function and decreased follicular synthesis of testosterone may in combination suppress de novo synthesis of estrogen. As shown by Zimmerman et al. [63] dams treated with DES had a 60% and 32% reduction in progesterone and estrogen levels, respectively, compared to the control group.

DES is no longer administered clinically for prevention of miscarriages but remains a useful compound for investigating the role of estrogen on fetal growth and development [70, 71]. Findings from DES studies are currently being used to better characterize the biological effects of other estrogenic compounds, including dietary estrogens [70–72] and BPA. The most commonly studied dietary estrogens during development are soy isoflavones because of their abundance in soy-based foods such as soy milk, tofu, miso, and soy protein [73]. Traditional Asian diets are rich in soy-based foods, and thus, many Asian populations consume between 15 and 40 mg of isoflavones a day [74–77]. At this level of exposure, there is no evidence that soy-based foods or isoflavones have adverse effects on human health. However, there are substantial differences in the form (fermented versus nonfermented), dose, and timing of isoflavone exposure between the traditional Asian and current Western diets [78]. Soybeans used for production of soy-based foods undergo some type of processing. Soybeans used in traditional Asian diets undergo germination, cooking, roasting, and fermentation while those used in Western diets employ more modern methods such as fractionation and extraction. The differences in soybean processing can affect the bioavailability of isoflavones and digestibility of soy protein through removal of indigestible sugars and inactivation of enzymes [79]. Studies show that fermented soy foods (i.e., miso and tempeh) are rich in aglycones that are more bioactive than the glycone form of isoflavones [79]. However, many unfermented soy foods available on the Western market can contain a substantially higher amount of total isoflavones (aglycone and glycone forms of isoflavone). For example, one soy burger contains 15–25 mg of isoflavones. A soy protein bar, often marketed as a meal replacement bar and sometimes used in weight management strategies, can contain 60 mg of isoflavones per serving. Consuming three to four servings of these soy foods a day in the Western World can exceed the level of isoflavone exposure reported in Asian populations (100–200 mg/day versus 15–40 mg/day). It is also possible that North Americans respond differently to soy foods than Asian populations because of their difference in timing of isoflavone exposure. Asian populations consume soy foods throughout their entire life, except for the brief neonatal period when most infants are breastfed [80, 81]. In contrast, in North America, soy is primarily consumed by infants fed soy protein formula or by health conscious adults eating a plant-based diet for general health and/or lowering risk of developing a chronic disease such as heart disease. Pregnant women consuming soy foods expose their fetus to isoflavones. Studies show that isoflavones are transferred readily across the placenta in humans and rodent models [82, 83]. Cord serum concentrations of genistein, daidzein, and equol correlate closely with maternal serum concentrations and isoflavones remain longer in fetal than maternal circulation [84]. Thus, depending on maternal diet, there is potential for a fetus to be exposed to substantial levels of isoflavones in utero.

 The spatial and structural arrangement of isoflavones is slightly different from that of estrogen, and thus, they can induce both estrogenic and nonestrogenic effects. Findings from two studies suggest that soy isoflavones may be able to alter fetal adrenocortical function [85, 86]. Feeding daidzein to late pregnant sows downregulates ER-*β* expression in the hypothalamus, which likely affects the regulation of fetal zone DHEA-S production [85]. In cultured human fetal adrenocortical cells, soy isoflavones (i.e., genistein and daidzein) decrease ACTH-stimulated cortisol production by inhibiting the expression of 21-hydroxylase (P450c21) but had no effect on DHEA-S production [86]. Further research is required to elucidate whether in utero exposure to soy isoflavone can alter DHEA-S production in vivo and whether there are other effects that may be subtle but nonetheless have lifelong implications to health.

## 10. The Effects of Environmental Estrogen on Fetal Adrenal Cortex May Be Mediated through Epigenetic Changes

 Although not fully understood, it has been hypothesized that estrogenic compounds during perinatal life can modulate epigenetic gene regulation, resulting in permanent effects that alter responses in later life and transgenerational inheritance. The most commonly studied epigenetic modification is DNA methylation, used by mammals to regulate gene transcription, chromosomal positioning, activation of X-chromosome, and gene imprinting. From a molecular perspective, DNA methylation is a term used to denote the attachment or substitution of a methyl group for a hydrogen atom on a cytosine residue. In vertebrates, DNA methylation depends on an enzyme known as methyltransferase which recognizes the CpG dinucleotides of various palindromic sequences and then catalyzes the transfer of the methyl group on the cytosine residue. As a result, modulators of DNA methylation are typically methyl donors or acceptors. However, estrogen-based compounds, which have been postulated to play a role in DNA methylation, cannot donate or accept a methyl group. Instead, it is believed that estrogenic compounds are able to enhance histone acetylation through interaction with ERs and that this interaction may open up the methylation region [87]. When this happens, methyl donors and cofactors including folate, choline, and SAM may stimulate DNA methylation [88].

 Fetuses are exposed to high concentrations of folate during early stages of development because mothers are advised to consume folate supplements during pregnancy to lower the incidence of spina bifida [89–91]. As a result, the combination of folic acid with high concentrations of circulating estrogens during early stages of development may cause an additive or synergistic effect on DNA methylation [88]. These changes in DNA methylation patterns have the potential to induce both beneficial and adverse effects on the developing genome. Future research is required to confirm whether early exposure to environmental estrogens can alter the plasma estrogen profile enough to induce long-term programming effect on the developing fetus.

To date, only one study has investigated whether soy isoflavones can alter DNA methylation patterns [88]. This study found that exposure to genistein resulted in hypermethylation of six CpG sites in the agouti mouse genome, which decreased the incidence of adult-onset obesity. ER-*α*, which has been shown to play a role in adrenal gland development and function, has at least eight promoters that contain CpG islands and are thus tightly regulated by DNA methylation [92]. Thus, it is possible that by regulating DNA methylation isoflavones as well as other environmental estrogens may be able to modulate adrenal gland growth and development.

## 11. Conclusion

The fetal adrenal cortex, together with the placenta and the maternal adrenal cortex, forms a unique fetomaternal endocrine system that regulates estrogen production during development. To date, notable progress has been made in understanding the embryogenesis and morphology of the fetal adrenal gland, the role of steroidogenic enzymes and nuclear receptors in differentiation of cortical zone function, and the process by which estrogen regulates its own production in the fetal adrenal cortex. However, there is still much work to be done in characterizing the effects of in utero exposure to environmental estrogens on fetal adrenal gland development and function. The available evidence suggests that exposure to environmental estrogens such as DES and soy isoflavones can reduce cortisol and DHEA synthesis in the fetal adrenal cortex as well as decrease placental estrogen production. The decrease in placental estrogen production during sensitive stages of development has the potential to alter gene transcription and DNA methylation patters of cells, suggesting that environmental estrogens may be able to induce epigenetic changes in the developing fetus.

Future studies on this topic should examine whether exposure to environmental estrogens can provide a causal mechanism to explain some of the endocrine-mediated adult-onset diseases. By integrating current knowledge of developmental biology with concepts of endocrine regulation, future studies will be better directed into uncovering the disruptions induced by environmental estrogens on fetal adrenal gland development and function.

## Figures and Tables

**Table 1:** The effects of estrogen on the mother and the fetus.

*Estrogen in maternal circulation:*	
(i) Enhances myometrial activity	
(ii) Soften collagen fibers in the cervical tissue	
(iii) Promotes myometrial vasodilation	
(iv) Increases uterine blood flow	
(v) Increases production of insulin-like growth factors (IGF-I/II) and binding proteins	
(vi) Promotes growth of the uterus, vagina, and breast	
(vii) Increases pituitary secretion of prolactin	
(viii) Increases sensitivity of the maternal respiratory center to carbon dioxide	
(ix) Stimulates synthesis and turnover of phospholipids	
(x) Increases serum binding proteins and fibrinogens to decrease plasma proteins	
(xi) Increases the sensitivity of the uterus to progesterone in late pregnancy	
(xii) Helps to control behavior including fatique, forgetfulness, poor concentration, as well as mild mood changes including irritability and depressed mood	
(xiii) Regulates salt and water retention	

*Estrogen in fetal circulation: *	
(i) Helps to maintain chemical levels in the bloodstream to achieve intrauterine homeostasis, which is the state of stability within the body	
(ii) Promotes maturation of fetal organs	
(iii) Regulates the fetal neuroendocrine system that controls reaction to stress, digestion, immune function, mood and emotion, sexuality, energy storage, and/or expenditure	
(iv) Regulates timing of parturition	

## Abbreviations

ACTH: Adrenocorticotropic hormone
CRH: Corticotrophin-releasing hormone
DHEA: Dehydroepiandrosterone
ER: Estrogen receptor
DHEA-S: Dehydroepiandrosterone-sulfate
P450c17: 17*α*-hydroxylase
HSD: Hydroxysteroid dehydrogenase.
